# Automatic thyroid nodule recognition and diagnosis in ultrasound imaging with the YOLOv2 neural network

**DOI:** 10.1186/s12957-019-1558-z

**Published:** 2019-01-08

**Authors:** Lei Wang, Shujian Yang, Shan Yang, Cheng Zhao, Guangye Tian, Yuxiu Gao, Yongjian Chen, Yun Lu

**Affiliations:** 1 Department of Gastrointestinal Surgery, The Affiliated Hospital of Qingdao University, No. 1677 Wutaishan Street, Qingdao, 266555 Shandong Province China; 2 Hisense Medical Equipment Co. Ltd, 399 Rd. Songling, Dist. Laoshan, Qingdao, 266000 Shandong Province China; 3 Department of Ultrasound, The Affiliated Hospital of Qingdao University, No. 1677 Wutaishan Street, Qingdao, 266555 Shandong Province China; 4 Shandong Key Laboratory of Digital Medicine and Computer Assisted Surgery, Qingdao, 266003 Shandong Province China; 5 Qingdao University, No.308 Ningxia Road, Qingdao, 266071 Shandong Province China

**Keywords:** Artificial intelligence, Thyroid nodules, Ultrasound, YOLOv2 neural network, Computer-aided diagnosis systems

## Abstract

**Background:**

In this study, images of 2450 benign thyroid nodules and 2557 malignant thyroid nodules were collected and labeled, and an automatic image recognition and diagnosis system was established by deep learning using the YOLOv2 neural network. The performance of the system in the diagnosis of thyroid nodules was evaluated, and the application value of artificial intelligence in clinical practice was investigated.

**Methods:**

The ultrasound images of 276 patients were retrospectively selected. The diagnoses of the radiologists were determined according to the Thyroid Imaging Reporting and Data System; the images were automatically recognized and diagnosed by the established artificial intelligence system. Pathological diagnosis was the gold standard for the final diagnosis. The performances of the established system and the radiologists in diagnosing the benign and malignant thyroid nodules were compared.

**Results:**

The artificial intelligence diagnosis system correctly identified the lesion area, with an area under the receiver operating characteristic (ROC) curve of 0.902, which is higher than that of the radiologists (0.859). This finding indicates a higher diagnostic accuracy (*p* = 0.0434). The sensitivity, positive predictive value, negative predictive value, and accuracy of the artificial intelligence diagnosis system for the diagnosis of malignant thyroid nodules were 90.5%, 95.22%, 80.99%, and 90.31%, respectively, and the performance did not significantly differ from that of the radiologists (*p* > 0.05). The artificial intelligence diagnosis system had a higher specificity (89.91% vs 77.98%, *p* = 0.026).

**Conclusions:**

Compared with the performance of experienced radiologists, the artificial intelligence system has comparable sensitivity and accuracy for the diagnosis of malignant thyroid nodules and better diagnostic ability for benign thyroid nodules. As an auxiliary tool, this artificial intelligence diagnosis system can provide radiologists with sufficient assistance in the diagnosis of benign and malignant thyroid nodules.

## Background

Thyroid cancer is the most common malignancy in the endocrine system, with an ever-increasing incidence [1–3]. Ultrasonography is the first choice for the examination of thyroid nodules. Ultrasound and fine needle aspiration biopsy (FNAB) examinations are commonly employed for the diagnosis of benign and malignant thyroid nodules. For thyroid nodules that are present, suspicious, or accidentally discovered by other imaging examinations, thyroid ultrasonography should be actively pursued [4]. The interpretation of ultrasound results primarily depends on the knowledge and experience of the radiologists, with profound subjectivity. A sharp increase in the number of patients has caused a significant increase in the labor intensity among radiologists and a reduction in the average diagnostic time spent on each case, which affects its diagnostic outcome [5].

The development of deep learning technology enables image recognition techniques to detect target areas in an image while classifying the detected target features, which is similar to the diagnostic process of imaging radiologists and provides a completely new idea for solving the previously mentioned problems. The deep learning algorithm developed by scientists from Stanford University has a comparable accuracy to that of dermatologists for identifying skin cancer [6]. The accuracy of artificial intelligence in the diagnosis of lung cancer, breast cancer, prostate cancer, and esophageal cancer in image recognition has surpassed that of experienced radiologists [7–10]. In recent years, computer-aided diagnosis (CAD) of thyroid nodules has rapidly developed and can effectively reduce errors caused by subjective factors and assist radiologists in rapid and accurate diagnosis [11]. However, these CAD systems generally need manually processed images in which areas of interest are manually labeled, which renders them clinically incompatible. Ma et al. [12, 13] proposed a hybrid method to classify thyroid nodules, which is a fusion of two pretrained CNNs. Ma et al. [14] also employed a deep CNN to automatically segment thyroid nodules from ultrasound images. The experimental results demonstrate the potential clinical application of the new method, but a clinical application has not been performed.

YOLOv2 is the algorithm published by Joseph Redmon and Ali Farhadi on CVRP in 2017. As the recognized deep neural network in the field of artificial intelligence, real-time analysis and efficiency are the most prominent features and advantages of YOLOv2. We aimed to establish an automatic recognition and diagnosis system for thyroid images via deep learning based on the YOLOv2 neural network and achieve real-time and synchronous diagnosis during ultrasound examination to aid radiologists in diagnosing benign and malignant thyroid nodules.

## Methods

### Establishment of an artificial intelligence automatic image recognition and diagnosis system

Traditional CAD systems for thyroid nodule image recognition require manual labeling of an area of interest and the identification of malignant and benign lesions via classifier(s). In our system, a target detection-based network framework is employed to enable the simultaneous identification of the location and type of thyroid nodule without requiring the selection of the areas of interest in advance.

**Fig. 1:**
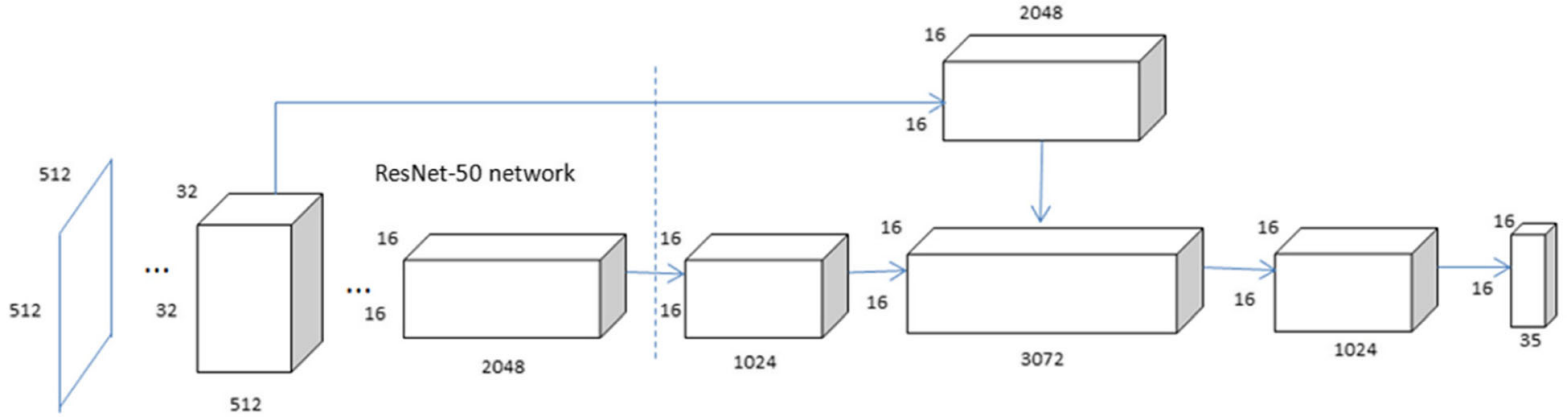
Improved network structure of the YOLOv2 network

Our system is an end-to-end detection network that is based on YOLOv2 [15] in which the Resnet v2-50[16] network and YOLOv2 are integrated. The last pooling layer and full connection layer of the deep residual network are removed, and the deep feature map of the network is fused with shallow features to obtain better fine-grained features. To reduce the computational load, a dimension reduction was performed by adding a convolutional layer of 1 × 1 × 1024 after the output of the deep residual network. For the input image, the bounding boxes and the probability at which each bounding box belongs to its class are obtained. In addition, this structure enables fast operation, which facilitates the real-time processing of the input image. The improved network structure of the YOLOv2 network is shown in Fig. 1.

To prevent the model from converging to the local optimum, the K-means clustering method was employed to analyze the data set to determine the number and size of the prior predictive frame. A total of 2450 benign nodules and 2557 malignant nodules, which were obtained from the ultrasound images of patients of different ages and genders, were collected and labeled as training data. To train the network model, a transfer learning method was employed. First, pretraining was performed on the visual object classes (VOC) data set to obtain the initial weights of the detection network. Second, iterative training was performed on the thyroid ultrasound image training set, by which the weights were fine-tuned to the optimal value. Last, the suspected lesion areas that were detected on the current image and the probability that the area is a benign or malignant lesion are output by the network.

The YOLOv2-based network is fast and capable of real-time detection. Compared with other detection networks [17, 18], the system established in this study can automatically identify the nodule location while determining whether the nodule is benign or malignant. This capability enables the thyroid nodule detection and recognition system to be embedded in an ultrasound imaging device to aid radiologists in making diagnoses. Our network can capture more fine-grained features of an image, enhancing the detection and recognition accuracy on small nodules. Compared with the traditional machine learning method, in which features are extracted and then trained with a classifier, our system uses a neural network to automatically perform feature extraction and recognition. Thus, the system is more robust and demonstrates excellent recognition ability on ultrasound images of different qualities acquired by different devices.

### Patient data

After training the system, 351 images with nodules and 213 normal images without nodules of 276 patients (53 males and 223 females, with an average age of 46.3 years and an age range of 20–71 years) who were hospitalized at our hospital (a tertiary class A hospital, regional medical center) from January to February 2018 were retrospectively selected. All selected patients underwent ultrasound examination in the hospital by senior radiologists (with more than 10 years of thyroid ultrasound examination experience) and subsequently underwent surgical treatment with pathological diagnosis results. The indications for surgery were detailed as follows: benign nodules with a diameter larger than 4.0 cm and malignant nodules with preoperative puncture pathological results. For nodules that were highly suspected of malignancy by ultrasound but do not meet the criteria of FNAB, FNAB was also recommended before further treatment. If patients refused FNAB and chose surgery, we would respect the patients’ willingness to perform the operation.

### Image acquisition and analysis

Ultrasound examination was performed by GE- logiq E8 (GE Healthcare, Milwaukee, WI), Philips iE Elite, and Philips iU22 (Philips Healthcare, Eindhoven, Netherlands) color Doppler ultrasound machines with a high-frequency linear array probe set at frequencies from 6 to 15 MHz, 3–11 MHz, or 5–12 MHz. The grading results of the senior radiologists according to the Thyroid Imaging Reporting and Data System (TI-RADS) were regarded as the diagnosis of the radiologists (a minimum grade of 4b the TI-RADS represents a high likelihood of malignancy, for which biopsy or surgery is recommended). The same image was input into the artificial intelligence system for interpretation, and the automatically labeled regions and discrimination between the benign and malignant thyroid nodules were regarded as the discrimination results of the artificial intelligence system.

One senior radiologist reviewed US images and drew bounding boxes to locate the nodules, while the artificial intelligence system drew the bounding box automatically. The region of interest box was assessed visually and classified into one of the following three categories: (1) excellent, the bounding box completely matched the nodule; (2) satisfactory, the bounding box volume was still representative of the nodule, and the maximum contour mismatch was visually estimated not to exceed 30%; and (3) poor, part of the nodule was enclosed in the bounding box, but the contour estimated mismatch was > 30% [19]. The first two categories (excellent and satisfactory categories) were regarded to be successful nodule localization.

### Statistical analysis

The difference in the interpretations of benign and malignant nodules from different diagnostic methods was tested using the chi-square test. Based on the pathological result, the sensitivity, specificity, positive predictive value (PPV), negative predictive value (NPV), and accuracy were calculated to evaluate the diagnostic abilities of the different diagnostic methods on benign and malignant thyroid nodules. At the same time, the areas under the receiver operating characteristic (ROC) curve and the 95% confidence interval (CI) were calculated. Additionally, the areas under the ROC curve for the artificial intelligence system and experienced radiologists were compared using the method of DeLong. *P* < 0.05 was considered statistically significant. All statistical analyses were conducted using the SPSS software version 19.0 (SPSS, Chicago, IL) and MedCalc for Windows (version 15.0; MedCalc Software, Ostend, Belgium).

## Results

In the selected 351 nodule images, the final diagnosis was described as follows: 109 benign (31.1%) and 242 malignant (68.9%). All nodules were confirmed by pathological findings after surgical resection. The distribution of patient demographic features is listed in Table 1. In the 109 benign nodules, four cases of Hashimoto’s thyroiditis, two cases of subacute thyroiditis, six cases of follicular thyroid adenoma, and 97 cases of nodular goiter (NG) were diagnosed. In the 242 malignant nodules, 240 cases of papillary thyroid carcinoma and two cases of medullary thyroid carcinoma were diagnosed.

**Table 1:** Summary of demographic feature

	Pathological findings		
	Benign	Malignant	*p* value
No. of patients	95	181	
Age, years, $\overline{\mathrm{x}}$ ± s	50.0 ± 10.6	44.3 ± 11.5	< 0.001
Sex			0.337
Male	15 (15.79%)	38 (20.99%)	
Female	80 (84.21%)	143 (79.01%)	
No. of nodules	109	242	
Size, cm	3.37 ± 1.81	1.17 ± 0.87	< 0.001
< 0.5	8 (7.34%)	23 (9.50%)	
0.5–1.0	13 (11.93%)	107 (44.21%)	
1.0–4.0	47 (43.12%)	106 (43.80%)	
≥ 4.0	41 (37.61%)	6 (2.48%)	

**Fig. 2:**
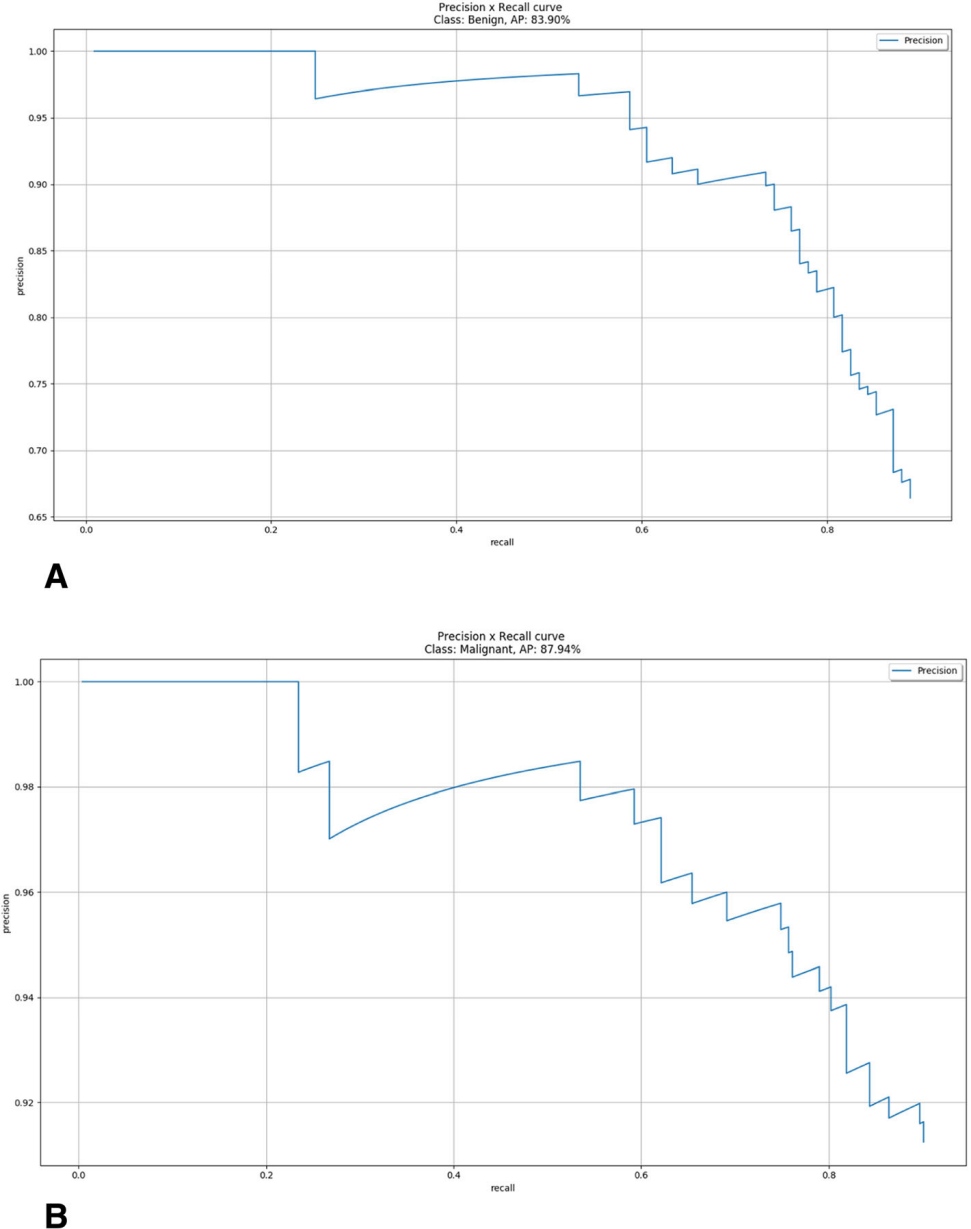
**a**, **b** We analyzed the performance of benign and malignant nodules target detectors using the Precision-Recall curve. In order to get the PR curve, we set the IOU between the bounding box and ground truth greater than 0.3 to be true positive, otherwise false positive. The AP value is used to evaluate the performance of the detector. The AP value can be obtained by calculating the area under the PR curve. As can be seen from the PR curve, the AP values of malignant and benign nodules were 87.94% and 83.90% respectively, and the average (mAP) of the two types of AP was 85.92%

**Fig. 3:**
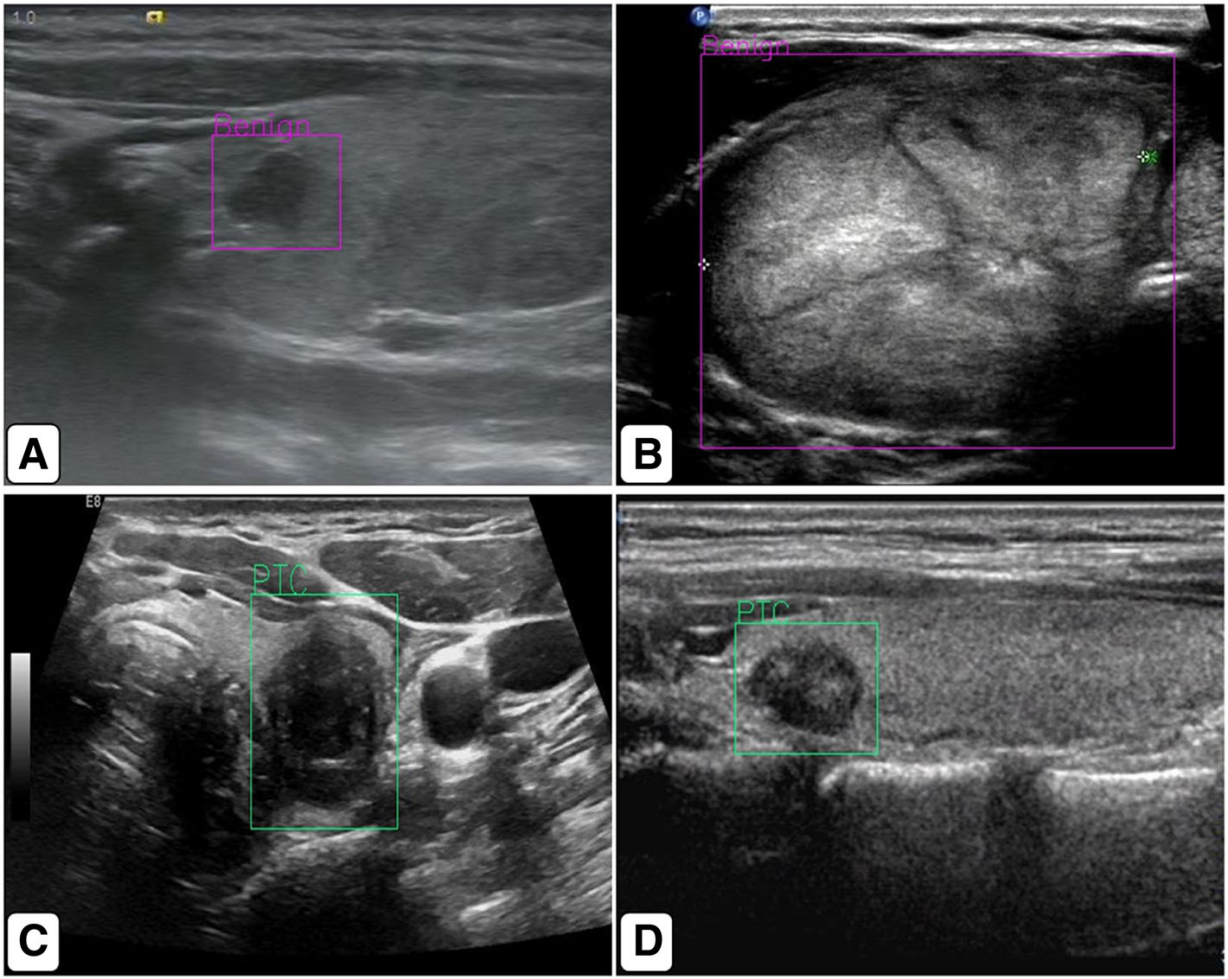
**a**, **b** Images of benign nodules. **c**, **d** Images of malignant nodules. The artificial intelligence automatic image recognition and diagnosis system can automatically identify thyroid nodules, label the nodules with rectangular frames, and discriminate the nodules and output

Excellent, satisfactory, and poor localization were observed in 76.4% (*n* = 268), 19.9% (*n* = 70), and 3.7% (*n* = 13) of nodules, respectively. For the benign thyroid nodules, excellent, satisfactory, and poor localization were observed in 71.6% (*n* = 78), 22.9% (*n* = 25), and 5.5% (*n* = 6) of nodules, respectively. For the malignant thyroid nodules, excellent, satisfactory, and poor localization were observed in 78.5% (*n* = 190), 18.6% (*n* = 45), and 2.9% (*n* = 7) of nodules, respectively. Successful nodule localization was significantly more frequent with malignant thyroid nodules than with benign thyroid nodules (*p* < 0.01). The performance of the thyroid nodule target detector is shown in Fig. 2a and b. The artificial intelligence automatic image recognition and diagnosis system was able to identify the position of thyroid nodules in 351 images, label the nodules, and distinguish between benign nodules and malignant nodules (Fig. 3). Of the 213 normal images without nodules, only two images were misdiagnosed to have nodules, in which one nodule was blood vessel and the other nodule was subcutaneous nodule.

**Fig. 4:**
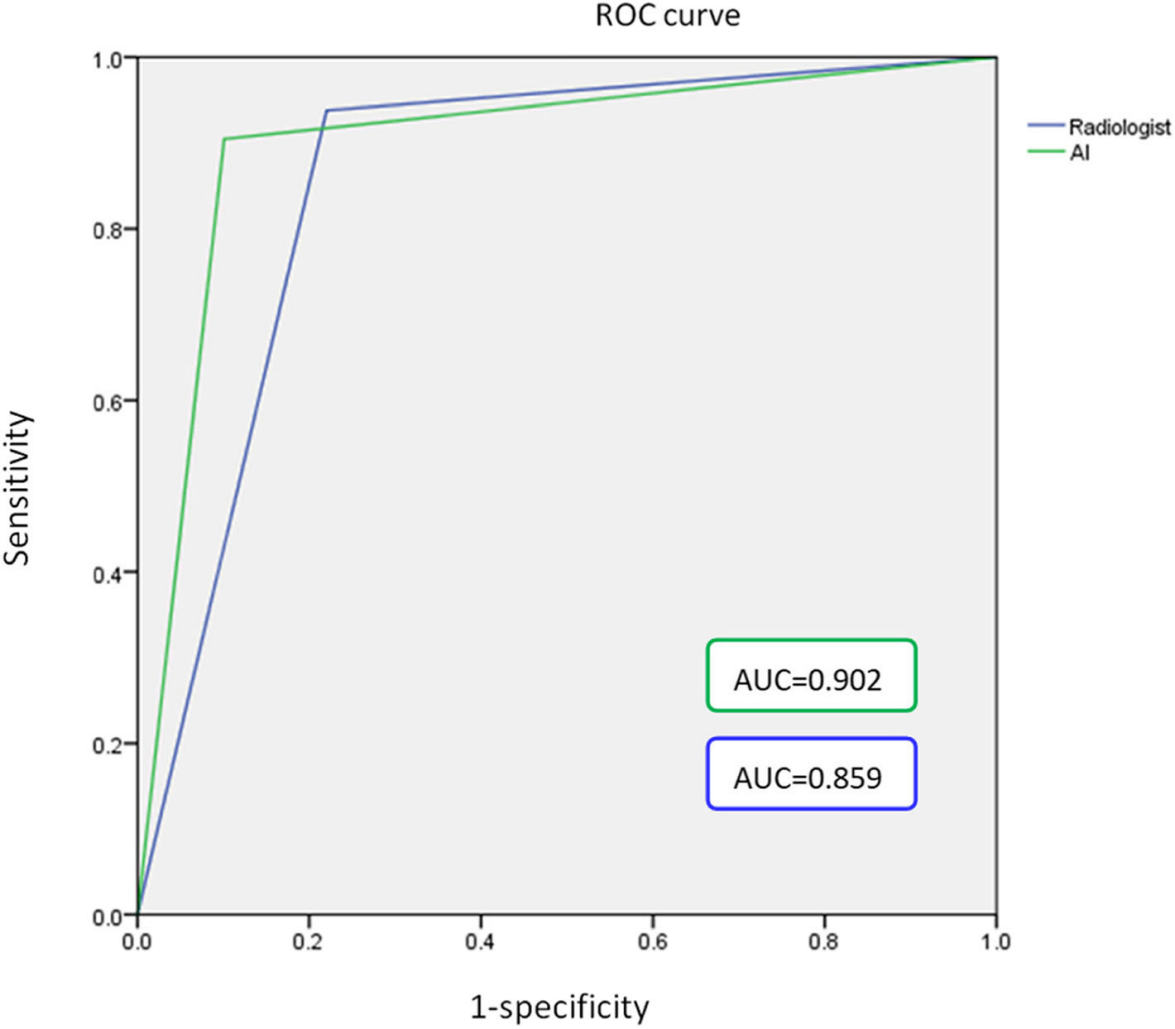
Areas under the ROC curve between the artificial intelligence system and radiologist were compared using the method of DeLong et al. The results indicated that the artificial intelligence system had a higher accuracy than radiologists (*p* = 0.0434)

Figure 4 shows the ROC curves of the artificial intelligence system and the radiologists in determining the benign and malignant thyroid nodules. The areas under the curve of the artificial intelligence system and the radiologists were 0.902 (95% CI, 0.866–0.931) and 0.859 (95% CI, 0.818–0.894), respectively (*p* = 0.0434), which indicates that the artificial intelligence system has a higher accuracy than the radiologists.

Table 2 summarizes the performances of the artificial intelligence system and radiologists in the diagnosis of thyroid cancer.

**Table 2:** Performances of the artificial intelligence system and ultrasound radiologists in diagnosing thyroid cancer

	Size, cm	AI system	Radiologist	*p* value
Sensitivity, %	< 1.0	88.46	93.08	0.284
	1.0–4.0	94.34	95.28	1.000
	≥ 4.0	66.67	83.33	1.000
	All	90.5	93.80	0.237
Specificity, %	< 1.0	57.14	14.29	0.009
	1.0–4.0	95.74	89.36	0.435
	≥ 4.0	100	97.56	1.000
	All	89.91	77.98	0.026
PPV, %	< 1.0	92.74	87.05	0.156
	1.0–4.0	98.04	95.28	0.446
	≥ 4.0	100	83.33	1.000
	All	95.22	90.44	0.053
NPV, %	< 1.0	44.44	25	0.305
	1.0–4.0	88.24	89.36	1.000
	≥ 4.0	95.35	97.56	1.000
	All	80.99	85	0.477
Accuracy, %	< 1.0	84.11	82.12	0.759
	1.0–4.0	94.77	93.46	0.809
	≥ 4.0	95.74	95.74	1.000
	All	90.31	88.89	0.621

*PPV* positive predictive value, *NPV* negative predictive value

**Fig. 5:**
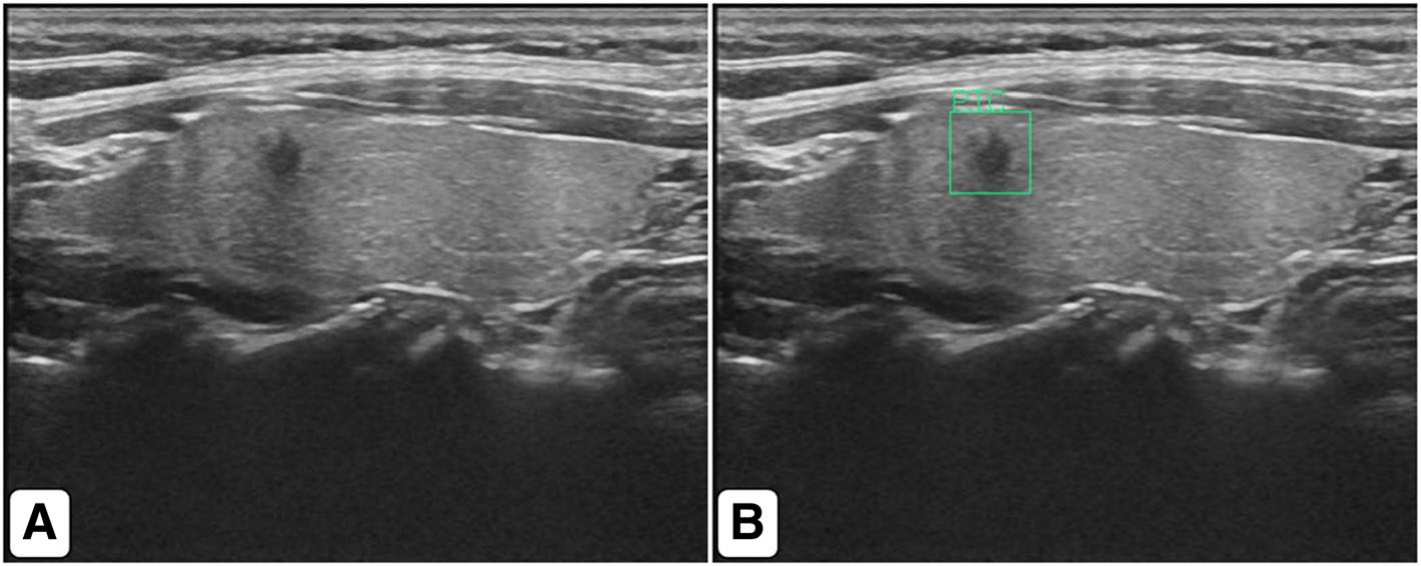
A 54-year-old woman with left thyroid nodule. **a** US images show a 0.3 cm × 0.2 cm solid hypoechoic nodule with macrocalcification in left thyroid gland. Radiologist diagnosed it as a benign nodule. **b** AI system diagnosed it as a malignant nodule. Histology confirmed diagnosis of papillary thyroid carcinoma

In 109 images of benign nodule cases, 85 cases (77.98%) were correctly identified by the radiologist, whereas 98 cases (89.91%) were correctly identified by the artificial intelligence system. The specificity of the artificial intelligence system and experienced radiologists in the diagnosis of thyroid nodules less than 1.0 cm in diameter was statistically significant (*p* = 0.009), indicating that the artificial intelligence system has better benign nodule diagnostic ability than radiologists (Fig. 5).

In 242 images of malignant nodule cases, 227 cases (93.8%) were correctly identified by the radiologists, whereas 219 cases (90.5%) were correctly identified by the artificial intelligence system; the two groups do not show any significant difference (*p* = 0.237).

## Discussion

The CAD system based on ultrasound images of thyroid nodules was proposed by Lim et al. in 2008 [20]. Compared with the CAD systems for the diagnosis of breast and lung malignancies, the CAD systems for the diagnosis of thyroid nodules have been rarely studied [21] and are outperformed by radiologists. Since the application of artificial intelligence neural networks to CAD systems, multiple studies have reported the drastically improved accuracy of CAD systems—and are as high as 98.3% [22–24]. Ma et al. [12, 13] proposed a hybrid method to classify thyroid nodules, which was the first attempt to use CNNs for detection of thyroid nodules. However, these systems were independently developed by the research team without the involvement of clinicians, which renders them unable to reflect the actual clinical situation and inapplicable to clinical diagnoses. In this study, according to the needs of actual clinical practice, an intelligent diagnosis system was established and the practicability of the artificial intelligence platform was verified. The sensitivity, PPV, and accuracy of the artificial intelligence system on the diagnosis of malignant thyroid nodules were 90.5%, 95.22%, and 90.31%, respectively, which were comparable to those of the radiologists (93.8%, 90.44%, and 88.89%, respectively), and the differences between the two sets of findings were insignificant (*p* = 0.237). This result is especially important in a developing country, such as China. The 5-year survival rate of thyroid cancer is 98.2% in the USA [25] and 77.6% in European countries [26] but only 67.5% in China, which is primarily attributed to a large gap between eastern China and western China. The levels of diagnosis and treatment in the primary and secondary hospitals are uneven. In this study, the PPV, accuracy, and area under the receiver operating characteristic curve (AUC) of the established artificial intelligence diagnosis system were higher than those of experienced radiologists. Although this cannot effectively improve the diagnosis accuracy of experienced radiologists, it can effectively improve the diagnosis accuracy of young radiologists and radiologists from primary hospitals and shorten the growth cycle of the radiologists. An artificial intelligence diagnosis system only requires a short time to make a diagnosis. In reference to the diagnosis results of the intelligent diagnosis system, the diagnosis time of the radiologists can be effectively reduced, which eases the burden on the radiologists.

Slow progression and excellent prognoses have earned thyroid cancer a reputation as a “cancer of no death” in China and enable a variety of treatment options. The increased detection rate of thyroid microcarcinoma has not caused mortality changes, which has raised concerns about the overdiagnosis and overtreatment of thyroid cancer in the medical community [27, 28]. Professors from Kuma Hospital argued that active surveillance of thyroid microcarcinoma is more beneficial than surgery for patients [29]. Although preoperative puncture biopsy achieves pathological diagnosis of the disease and has a high safety and diagnostic specificity, the average diagnostic accuracy is approximately 83%. A significant proportion of diagnoses is false positive and may even require a second biopsy [30]. In this study, the specificity of the artificial intelligence diagnosis was 89.91% and the correct identification on benign thyroid nodules was (98/109), which shows a higher diagnostic ability of the artificial intelligence for benign nodules (*p* = 0.026) than the diagnostic ability of radiologists for benign nodules (77.98% and 85/109, respectively). Therefore, the radiologists can use the data from the artificial intelligence system to confirm the diagnosis of benign thyroid nodules and exclude the diagnosis of malignant thyroid nodules, which prevents unnecessary punctures or surgical treatment and benefits patients while saving social medical resources.

The use of ultrasonography for thyroid diseases has substantially improved the detection rate of thyroid nodules. Current treatment guidelines indicate that ultrasonography is the preferred method for assessing the risk of benign and malignant thyroid nodules [4, 31]. Among the acoustic features of thyroid nodules, five features, i.e., aspect ratio > 1, low echo or very low echo, solidness, boundary irregularity, and microcalcification, are defined as suspicious malignant features [32]. Previous studies of CAD systems for thyroid nodules were based on the malignant acoustic characteristics of the thyroid nodules. Yu et al. [17] established an ANN diagnostic model and determined that the accuracy, sensitivity, and specificity of the model for the data of 45 patients were 90%, 88.24%, and 90.91%, respectively. Chi et al. [18] employed a deep convolutional neural network to identify benign and malignant thyroid nodules for the data of 162 cases of local patients based on ultrasound images and determined that the accuracy, sensitivity, and specificity were 96.34%, 86%, and 99%, respectively. In these studies, cumbersome image preprocessing, complex computer analysis, and lengthy computation time are required, which renders them impossible for detecting thyroid nodules in real time. Yoo et al. [33] reported a CAD system that is capable of real-time detection with the accuracy, sensitivity, and specificity of 84.6%, 80%, and 88.1%, respectively. However, the region of interest still needs to be manually marked. In our system, the areas of interest are automatically labeled, and the benign and malignant nodules are identified with an accuracy, sensitivity, and specificity that are comparable to those of the previously mentioned studies. In addition, the artificial intelligence system established in this study achieves rapid diagnosis, enables real-time and dynamic inspection in ultrasound examinations, and has broad prospects for clinical applications. The artificial intelligence system has been embedded in the ultrasound imaging equipment produced by Hisense and is being verified.

This research has some shortcomings. First, the majority of malignant nodules were papillary thyroid carcinoma (240/242). The acoustic characteristics of papillary thyroid carcinoma, medullary carcinoma, or other malignancies, such as lymphoma, are substantially different [34]. Therefore, additional images of various types of thyroid malignancies are needed to train the system. Second, we only compared the performances of the artificial intelligence system and radiologists in diagnosing benign and malignant thyroid nodules. We did not evaluate how much the artificial intelligence system has enhanced the diagnoses of the radiologists. The artificial intelligence system is designed to aid the diagnosis, but is not designed to replace the radiologists. After completing the integration of the artificial intelligence system and ultrasound imaging equipment, we will assess the enhancement of the artificial intelligence system on the diagnosis ability of radiologists with different levels of experience. Third, this study is a single-center study conducted in the Affiliated Hospital of Qingdao University. Selection bias may exist; in the future, multicenter studies should be conducted to improve the database needed to train the artificial intelligence system. Thus, its diagnostic capability can be improved. Last, the system will perform autonomous learning on metastatic lymph nodes in the neck and improve its diagnostic capability by combining with the characteristics of thyroid nodules.

## Conclusions

The sensitivity, PPV, and accuracy of the artificial intelligence automatic image recognition and diagnosis system do not statistically differ from those of experienced radiologists in diagnosing thyroid malignancy, and the specificity of the artificial intelligence system is higher than that of radiologists. Therefore, the artificial intelligence system can help radiologists exclude a false-positive diagnosis (benign nodules that are considered to be malignant by the radiologists), and unnecessary puncture or surgery can be avoided. The real-time detection capability of the artificial intelligence system can help radiologists save diagnosis time, reduce labor intensity, and reduce the impact of subjective factors on diagnosis. Therefore, the established artificial intelligence system has substantial potential in assisting radiologists in diagnosing benign and malignant thyroid nodules. The clinical application value of the artificial intelligence system needs to be further evaluated in the actual clinical practice of radiologists with different experience levels after the completion of the integration of the system into ultrasound equipment.

## Abbreviations

AUC: Area under the receiver operating characteristic curve
CAD: Computer-aided diagnosis
FNAB: Fine needle aspiration biopsy
NPV: Negative predictive value
PPV: Positive predictive value
ROC: Receiver operating characteristic
